# Medical Statistics

**Published:** 1841-01-01

**Authors:** 


### MEDICAL STATISTICS.

I. Statistical Reports of the Health of the Navy, for the Years 1830, 1831, 1832, 1833, 1834, 1835, and 1836, in the South American, West Indian, North American, Mediterranean and Peninsula Commands. Ordered by the House of Commons to be Printed. 1840.

[Concluding Notice.]

II. Second Annual Report of the Registrar General of Births, Deaths, and Marriages, in England, with Appendices.

Our readers will do us the justice to admit, that we have encouraged, in every possible way, the publication of Statistical Reports. We have ever been convinced, and we still are so, of their utility, and, directly or indirectly, they will prove, we feel assured, the most powerful instrument we now possess for the advancement of our science.

It may be true that some enthusiasts push their application beyond its legitimate limits. But extravagance is always found to accompany the introduction of new means of eliciting truth, and cannot permanently derogate from their substantial value. It is the part of men of sense to accept all the good, and allow the evil, if such exist, to evaporate under the influence of reason and time. *They* may be trusted for setting these matters in their proper light, and establishing, at last, their real worth. We turn to the Reports before us.

### 1. Health of the Navy.

In our last Number, we *joined* our brave tars on the South American, West Indian, and North American Stations. We deferred to this occasion a trip with them to the Mediterranean. And who would not accompany them *there now?* Who would not sail with the gallant Napier, and encounter the Egyptian muskets and the Syrian dysentery, for the sake of such laurels as our blue-jackets have won for Old England at Beyroot, and Sidon, and Acre. Even while we landsmen

"Who lie at home at ease,"

are writing of the fevers and the fluxes that may seize on them, our countrymen are, we grieve to say, struck down by them, and the cannon of Acre are less feared than they.

### Mediterranean and Peninsular Commands.

*Year* 1830.—This command embraces the seas, and shores of the Mediterranean, and Gibraltar. It extends over less space than some other commands, comprises fewer degrees of latitude, and is less exposed to extreme difference in degrees of temperature; it does not include more than 12 degrees, those namely, from the 32d to the 44th north. As respects geographical position, therefore, the term " temperate" has been especially applied to it, and it has obtained high reputation for its influence on health. Yet the difference between the south and north shores, as regards temperature, is often great, especially in winter; the vicissitudes on the north coast are sudden, and violent.

The sirocco, south-east wind, blowing from the African continent, is singularly depressing, suddenly producing such languor, oppression, and feelings of feebleness, as neither its temperature, nor other appreciable quality can account for. It seldom continues to blow during many consecutive days. Its influence is most felt near the coast of Africa, but it is often powerful at Malta and Sicily, and sometimes reaches the north shores of the Mediterranean.

*Malta*, on account of its central situation, its arsenals, and the excellence of its harbours, is the principal naval station. During nine months of the year, the temperature is moderate, the weather being generally clear and fine, which, with the excellence of fresh meat, and vegetables procured there, produces highly beneficial effects on the health, comfort, and efficiency of the naval force employed in the Mediterranean. The other three months are hot, sometimes in a high degree; yet it has rarely proved very prejudicial.

Every anchorage in the circuit of the Mediterranean is visited, and more or less frequented, by ships of war. The parts most resorted to are Malta, Smyrna, various Greek, and Levantine islands, and Gibraltar, the extent to which they are occupied differing much at different periods.

The numerous ports, and places, embraced by this command differ greatly as to extent, exposure, and physical structure, and considerably in regard to external heat; they therefore act very differently on the health of ships' companies resorting to them; but, on the whole, and apart from some rare, but severe, epidemics which have affected some of them, their influence, combined with that of the sea climate, is very favourable.

The naval force employed on the coasts of Spain, and Portugal, is included in the Report for the Mediterranean. From the frequent interchange of portions of the force of the two squadrons, ships passing from the Peninsula to the Mediterranean, and *vice versâ*, it was found impossible to separate them, so as to show satisfactorily the power of each on health.

The mean number employed in the united squadrons during the year was 6,576.

The health of the naval force, during the year, was high. From all causes there were 59 deaths, being at the rate of considerably less than one dead out of every hundred men employed. In three instances death resulted from external injuries at sea: so that the rate of mortality from disease was 8·5 per 1,000; from all causes, nine per 1,000 of force, abroad. But seven invalids from the station died in home hospitals, making a total of 66 deaths on, and from, the station, which is in the ratio of 10 dead out of every 1,000 employed, both abroad, and at home, during the year. Of the other fatal cases, 43 were on board, 16 in foreign hospitals.

The total number of cases treated was large, viz. 9,305, giving the very high ratio of 1415 per 1,000 of force. Out of the total number, 319 were sent to hospitals for treatment, and 189 were invalided, 99 from ships, and

90 from hospitals; the invalided were in the ratio of 28·7 per 1,000 of force. Thus it appears, that, deducting from the total number of cases, 319 sent to hospital, 43 terminating in death, and 99 in invaliding, on board, 8,844 were treated successfully in the ships where they occurred.

Of all forms of idiopathic fever, and probably some symptomatic affections classed with them, there were 709 cases; of which 628 were continued, 34 remittent, and 47 intermittent; of the first, seven terminated in death; of the second, five; of the third, none; of the first, 23 were sent to hospital; of the third, two; of the second, none. Three of the 25 cases sent to hospital terminated fatally. The vessel that suffered most severely was the Mastiff, a small surveying vessel, five deaths from eleven attacks occurring in her, four from remittent, and one from continued fever. She was employed on the coast of Thessaly. The people, as must be in such service, were much exposed to the weather in open boats; it rained heavily at times, the mean temperature being 76°. In such circumstances, and being, it is presumed, on a marshy coast, remittent fever was to be expected.

There were 227 cases of inflammation of the lungs, and their investing membranes; of which 88 were sent to hospital, four terminated fatally at sea, and 25, in a chronic condition, were invalided. The number affected was large, the relative loss by death small.

Inflammation of the lungs, and of their membranes, especially the interior, lining the air-passages, is rather a prevalent form of diseased action in the Mediterranean, for which the nature of the climate, in regard to temperature particularly, sufficiently accounts. It is, as has been stated, very variable, the changes, especially on the north coast, being sudden, and often violent. When the wind shifts from south to north, and *vice versâ*, a fall or rise of 20 degrees in the thermometer will sometimes take place in a few hours.

However strange it may appear, it has been ascertained that bronchitis, which originates in the Mediterranean, and which often runs rapidly to a fatal termination there, may be arrested by moving the subject to England. A similar remark was made respecting the disease in South America.

Of less violent, but more dangerous diseases affecting the pulmonic tissue, there were considerable numbers. The phthisical cases, including hæmorrhages from the lungs, amounted to 53, viz. 36 of the former, and 17 of the latter. The latter is not necessarily a symptom of the former, nor of disorganization so great as to destroy life, though it certainly is so in a great many instances. It is therefore remarkable that none of these 17 cases proved fatal. It is also remarkable, that out of 36 cases designated phthisis, only four terminated fatally abroad, and one in hospital at home, though 14 were sent to hospitals abroad, and seven were invalided. There were two fatal cases of chronic catarrh in home hospitals; whether these cases ought to have been originally, and correctly, designated phthisis or not, cannot be ascertained; but even if they were, the loss from phthisis, particularly in relation to the number affected, would be singularly low. It can scarcely be doubted, that the term has been in some cases erroneously applied, and that less fatal diseases, such as chronic bronchitis, or pleuritis, have been mistaken for true tubercular phthisis; but be that as it might, it is certain that the loss from pulmonic disease was very small, and that the station maintained its reputation during the year, as far as the experience of the squadron was concerned.

Under the appellation of inflammation of the liver there were 75 cases, of which 17 were sent to hospitals for treatment, three were invalided, and two terminated fatally.

From all the cases of inflammatory disease of the alimentary organs, there was only one fatal in its termination.

There were 35 cases of small-pox in the Blonde, and one in the Wasp, at Malta, where the disease was prevalent among the citizens. In the Blonde it was generally mild, the eruption having little tendency to confluence, and there being little secondary fever. No one terminated fatally. It is stated that the vaccine cicatrix was well marked in all the crew, except six individuals, to whom the virus was immediately communicated. Besides these 35 cases, two were sent from the Asia to Malta hospital, both described as small-pox by the surgeon of the ship, and both fatal. One had, seemingly, had the small-pox previously, the other had apparently not been vaccinated.

The dysenteric cases amounted to 120; the most of them were slight, and easily cured. There were three fatal cases; three were sent to hospital, and one was invalided.

There were 145 cases of common cholera. Out of the whole number, one man died in his ship, the Blonde, and one was sent to hospital, the remaining 143 having recovered in their respective ships.

Though there were only four cases of delirium tremens, two terminated fatally.

Cases of venereal disease were numerous. There were 245 of syphilis, 291 of gonorrhœa and stricture, and 62 of bubo and swelled testicle. Though the last had not all a venereal origin, it is probable that the majority of them had. Glandular disease is common in the Mediterranean, and buboes frequently occur without any trace of a venereal cause.

The cases of common ulcer were also numerous, viz. 532; but they were all, except 11, sent to hospital for treatment, and four invalided, cured in the ships where they occurred.

The wounds, accidents, and external injuries of all kinds, amounted to the large number of 1,729 cases; of which 36 were sent to hospital for treatment, four were invalided, and three terminated fatally at sea. In a great majority of instances they were very slight, and speedily cured. Of the cases sent to hospital, three terminated fatally.

Such was the sickness, such the mortality, on this command, during the year 1830. We need not enter so much into details in the succeeding years, but rather content ourselves with indicating their differential characters. The chronicle of the first will have conveyed a good notion of the general salubrity or otherwise, of the Command.

*Year* 1831.—There was less death on the station than there was even in the preceding year, but there was considerably more in home hospitals, from disease contracted on it; so that the total mortality resulting from service, in the united squadrons, was a little more in 1831, than it was in 1830.

The mean numerical force of the year was less than that of the preceding year, being 5,714. The number of deaths during the two years 1830 and 1831, was the same, viz. 60. The ratio of mortality was, however, a little lower in the first than the last; in that it was, from all causes, on the station, and in home hospitals, at the rate of 10, in this it is, at the rate of 12·3 per 1,000 of force.

During the year under consideration, there were 50 deaths, from all causes on the station, 31 of which were at sea, and 19 in hospitals, being in the ratio of 8·8 per 1,000 dead of the employed. But of these 50 deaths, 10 were from external injuries; so that the ratio of mortality from disease, on the station, was little more than seven per 1,000 of force. Of the invalids sent to home hospitals, 20 died, making a total of 70 deaths, from all causes, at home and abroad, being, as has been stated, in the ratio of 12·3 per 1,000; and from disease, separate from external injury, 10·5 per 1,000 dead of the employed. The points in which the two years principally differ, are the greater mortality in home hospitals, and from external injuries in 1831, than in 1830. In 1830 there were only seven deaths in home hospitals, and six from external injuries; in 1831 there were 20 of the former and 10 of the latter. It often happens, however, when patients die in home hospitals from foreign stations, that death takes place the year after they were invalided abroad, which, so far, would lead to erroneous conclusions respecting a single year. In determining the absolute mortality resulting from the disease of a foreign station, by adding to the deaths on it, those which occur in home hospitals, from it, it is therefore essential to take the result of a series of years, little precise information being deducible from those of one year.

The total number placed on the surgeons' list during the year amounted to 8,883, being at the rate—higher even than that of the preceding year—of 1554 per 1,000 of force. Of the total number, 411 were sent to foreign hospitals, where 19 terminated fatally. The number sent to, and the number which terminated fatally in, foreign hospitals, are higher, each by about one-third, than in the preceding year, though the ratio of mortality on the station is lower. Sending seamen and marines to hospital is affected greatly by opportunities. When disease occurs far out at sea, or in harbours where there are no British hospitals, however severe or prevalent it may be, it must, of course, be treated to its termination on board.

Invaliding was resorted to in 163 instances, 80 on board, and 83 in foreign hospitals; the rate therefore at which numerical force was reduced through this medium was 28·5 per 1,000, being, within a very small fraction, the same as in the preceding year.

Thus, as 411 were sent to hospital for treatment, 80 were invalided, and 31 died in ships, 8,361 were cured on board.

A rather curious account is given of fever on board the Pallas. She sailed in December 1830 from England, for the Mediterranean, touching at Lisbon, proceeding to Malta, thence to Salamis and Spezzia, and then returning to Malta; at each of these places she remained only a few days. She finally left Malta for England on the 2d of February, 1831, having embarked there 200 men of the 90th regiment, 41 women and children, and 15 invalids from the squadron. From the time she left England till her return, the weather had been generally fine, for winter; the range of the thermometer was small, the lowest noted being 57°, the mean 63°. When off Sardinia, on her return passage, fever appeared, on the 8th of February, and, before the 21st, had affected 100 persons, when it suddenly and entirely ceased. It attacked equally the men, boys and officers of the ship; it attacked, but less extensively, the soldiers and invalids; it did not attack the women or children. What was the cause of this disease?—why were the women and children exempted from such a sudden and rapid epidemic, living as they did in the same place with those who were attacked by it? The wind, after leaving Malta, till the 8th, had been from the south and west, when it suddenly shifted to the east. The disease would appear to have been one of the few febrile disorders, prevailing epidemically, which owe their origin to a purely atmospheric cause, at sea. The reason why the people belonging to the ship suffered more than the male adult passengers, while the women and children escaped, probably depended on exposure, and the degree of it, to the direct influence of the wind. The ship's company, officers, men, and boys, were necessarily much on deck, while the soldiers and invalids were below, at least at night, where the women and children were almost constantly.

Idiopathic erysipelas, with tendency to gangrene, is not now, though it once was, a prevalent disease in the navy; it is therefore a striking feature in the mortality of this year in these commands, that nine deaths resulted from it, seven at sea, and two in hospitals. Of the seven deaths at sea, four were in the Prince Regent, out of 26 attacks. She had been employed a considerable time in the Tagus, where the disease prevailed rather extensively. Sometimes a slight scratch or contusion appeared to call into action the erysipelatous force with which the system was charged, or to open the way for the operation of the specific cause of the disease; at other times, or rather in other instances, the first local symptom was a small furuncular swelling, independent of external injury. There was perhaps a prevailing disposition to the disease, as well as a peculiar cause for it.

The other three fatal ship's cases were in the Ganges, out of eight attacks, which occurred in her at sea, on a passage from Malta to England, and happened, in fact, in the beginning of 1832, though the return is dated in 1831. They occurred nearly at the same time, and were strictly idiopathic, no external exciting cause appearing in any of them. For such an effect there was a peculiar, probably diffused cause in the ship; and that no more cases occurred would appear to have depended on the low state of susceptibility of the crew generally to the operation of that cause. What the cause of the disease is, and why, as sometimes happens, it shall prevail in one ship, and not touch another close to her, is a subject of interesting inquiry.

The ulcerative cases were in the ratio nearly of the preceding year, amounting to 549; of which 35 were sent to hospital for treatment, and four were invalided. Of the total number 137 were in the Ganges, 18 of which were sent to hospital. Most of them were in September, the hottest month of the year, in Malta harbour, the subjects being generally marines. The disease during that month, when 81 cases occurred, presented a very uniform appearance. If affected the feet, close to the toes or ankles, the first symptom being a livid spot, which did not extend beyond the size of a penny-piece, and to which extent the integuments sloughed. It is stated by the surgeon, that the disease was not contagious, but "depended on some peculiar state of cuticular irritability, superinduced by the condition and temperature of the atmosphere." It should perhaps have been added, that some morbific agent, some local atmosphere existed in the ship—her own peculiar product—which contributed to the origin, and continuance of the disease, if it did not alone cause it; for it is remarkable that, when cases were sent to hospital, they speedily improved, the sores assuming a more healthy aspect within the first twenty-four hours. Besides, it does not appear that ulcer of the same kind prevailed in other ships at the same anchorage.

*Year* 1832.—While the ratio per 1,000 of mortality from all causes on the station was 8·8 in 1831, it is 10·1 of force in the present year; but the total mortality of the former is higher by a fraction, on account of a larger proportion of deaths in home hospitals, from disease originating on the station. Again, though the number of deaths from external injuries was, at sea, the same in the two years, viz. 10, the reduction of mortality from disease, on the station, was considerably less, by them (accidents) in 1832 than it was in 1831; in 1831 the ratio per 1,000 force of mortality, from disease abroad, was little more than seven; in 1832 it was 8·5; the difference between the two years in this respect depending on difference in the number employed. Other differences in the details of the two years will be noticed afterwards.

The mean number employed during the present year was 6,634, being an increase of 920 on the active force of the preceding year. The total number of sick and hurt was 7,659, being in the ratio of 115·4 per 1,000 of the employed, while in the preceding year it was 155·4 per 1,000; making a reduction of a third nearly of sick and hurt in favour of the present year. Yet the total mortality of the two years, including the deaths in home hospitals, was almost exactly the same, and abroad considerably more in 1832 than in 1831; showing, with many like cases, how uncertain the relation between sickness and mortality often is.

Thus, it happens that a fever becomes suddenly epidemic on board ship, but scarcely, if at all, affects life, as happened in the Pallas, and other ships in the Mediterranean, in 1831. It also happens frequently when one disease prevails epidemically, that other diseased actions are suspended, or are less frequent, the master-malady, so to speak, having the power of resisting the attacks of other occasional diseases; and thus it happens that epidemics which have little destructive tendency, and are skilfully managed, though they prostrate great numbers, and create alarm, do not augment mortality. Even in severe epidemics, it is often matter of surprise, when they and the consternation which they caused are past, on comparing the total mortality resulting from them, and other diseases, with the mortality in equal periods when no such epidemic existed, the periods being considerable and the masses large to which the comparison is applied, that the increase of mortality during the epidemic period is so little as it is found to be.

Of the total number placed on the medical lists, 284 were sent to hospitals for treatment, and 147 were invalided; the proportion of both, especially of the first, was considerably less than in the preceding year. It was in the ratio of 42·9 per 1,000 of force, whereas in 1831 the ratio of cases sent to hospital was 71·9 per 1,000; and while the ratio of invalided in 1831 was 28·5 per 1,000 of force, abroad, in 1832 it was 22·2 per 1,000.

There were 242 cases of inflammation of the lungs, and their investing membranes, of which nine, all at sea, ended fatally; 21 were sent to hospitals for treatment, and two, in a chronic state, were invalided; there were two deaths at home. Of the total number of attacks, 55 were in the Caledonia, three of which terminated fatally. At the same time she had a great number (211) cases of catarrhal disease, many of them so severe as to approach the character of pulmonitis. All the cases occurred within eight months, the time between her arrival on the coast of Portugal and the close of the year. It is not easy to account for such a number of severe pulmonic affections in this ship—a ship of the line of the largest class. She was employed the greater part of the time in the Tagus, and off the coast of Portugal, where the atmospheric heat was generally rather high, and steadily so; a number of cases occurred before she sailed from England. The lowest degree of heat noted in England was 41, the highest on the coast of Portugal 75; but between the extreme points there was a lapse of weeks, and it appears to have risen and fallen very gradually. It was, therefore, generally mild, and as uniform as it almost ever is in such latitudes. There were none of those sudden, and violent changes of weather, especially in respect of heat, to which pectoral inflammation generally, and it would appear reasonably, is ascribed. How, then, is this prevalent form of diseased action to be accounted for? It is stated by the surgeon of the Caledonia that no other ship on the station had such a large proportion of disease. It is further stated, that she was in a very damp state, from frequent washing of decks, to which he is disposed to attribute much of the pulmonic disease, as well as other forms of disease, particularly erysipelatous. It appears that in June the practice of washing decks was less frequent than it had previously been, but the degree of diminution is not noted, nor whether it was increased, or lessened afterwards; the means are therfore imperfect for tracing the relation between washing decks, and disease. But, apart from this case, there is no reason to doubt that it is many ways, and often to great extent, prejudicial; for in addition to the sudden reduction of temperature resulting directly from it, when frequently applied, the decks are never dry, in the proper and healthy sense of the word, whatever means may be adopted for the purpose.

We look on this as a very interesting fact. The practice of excessive washing would really seem to be a most noxious one. We are confident that it has done, and is doing great mischief in our hospitals ashore. Contrast what occurred in the Caledonia with what happened in another ship on the same station.

The Asia, of 84 guns, was, at the same time, and during four previous months, employed in the Tagus, or near it, having had during the year only nine cases of pulmonic inflammation, and three of catarrh, all of which terminated favourably on board. As has been stated, the Caledonia, in the same position, and during eight months of the same year, had 55 cases of pulmonic inflammation, and 211 of catarrhal disease, so severe as to resemble fever complicated with pneumonia. It may be further stated, that, on comparing the general results of disease in the two ships, during the periods specified, while 12 were sent to hospital, and four invalided from the Asia, 38 were sent to hospital and 16 invalided from the Caledonia; and that there was only one fatal case in the former, while there were eight in the latter ship.

Eight deaths in as many months in a ship having the complement of the Caledonia, do not certainly make a serious loss of life, being in the ratio of little more than one and a quarter per cent. per annum of the employed, but it is large by comparison with the loss sustained in the Asia. The comparison between the number of pulmonic attacks in the two ships is still more striking, and in this point lies the chief interest; for to difference in some thing, or things within the two ships, as it regards the number affected, must the difference be traced. It may be fairly presumed that the cause of difference in these cases should be sought for in the different means of cleaning, and ventilating adopted, though in many cases the component parts of the ships themselves should enter largely into the account. It is unnecessary to point to the important uses derivable from such an inquiry, or to assert, if strictly carried out and acted on, that it would conduce greatly to increase of health.

In the Caledonia, again, there were 74 cases of ulcer, but all of them, with one exception, were cured on board. There the ulcerative process was, perhaps, associated with the erysipelatous, which had some prevalence in her; there is sometimes identity in cause, with considerable difference in modes of development.

*Year* 1833.—The mortality of this year is more than that of the three preceding ones, though the increase on the first is inconsiderable, and on the two following, in which it was the same, trifling. In the year under notice, from all causes, and including the deaths, in home hospitals, from disease manifesting itself within the limits of the commands, it was in the ratio of 13 per 1,000 of force. From all causes on the stations it was in the ratio of 10·5 per 1,000 of force; and from disease on the foreign stations, separate from external cause of death, the ratio of mortality was under nine per 1,000 of active force. This, when it is considered that malignant cholera was added to the ordinary causes affecting life, and that it occasioned a considerable portion of the total loss, is a very low rate of mortality, and goes far, in connexion with preceding Reports, to show that the climate of the Mediterranean, and of the adjacent sea and harbours, is generally, and apart from occasional rare epidemics, highly favourable to the health of sailors.

The force of the united squadrons in the Mediterranean, and on the coasts of Portugal and Spain, in 1833, was large, comprising 51 vessels of all descriptions, eight of which were ships of the line, three of them three-deckers; the number of men and officers employed being 7,836.

The total number of sick and hurt was 10,274, being in the ratio of 1,311 per 1000 placed on the medical lists, of the employed. This ratio is certainly not low in itself, but it is worthy of remark, that it is considerably lower than it was it 1830 and 1831, though that of mortality is higher.

We turn to some particular facts.

Of the whole number, viz. 299 cases, of remittent fever, a very large proportion, 243, were in the Asia. The disease prevailed principally between April and September, the Ship being at anchor in the Tagus. It was, in most cases, not violent in impression, and short in duration, seldom lasting more than four days; only one case terminated in death. The surgeon believes that it had a miasmatous origin, derived from the contiguous coast. Forty-two of the 66 remaining cases of this form of fever occurred in the Donegal. She also was in the Tagus, and in her, as in the Asia, the disease was generally not violent, aud of short duration. In both cases the surgeons are disposed to attribute the fevers to some modification of the agency which, at the same time, occasioned cholera in some of the ships, and at Lisbon, in which city it destroyed a large number in a short time. This conclusion seems to have been drawn chiefly from the circumstance of the alimentary mucous lining being in an irritable state, with frequent evacuations, sometimes resembling those in cholera.

This looks like those attacks of diarrhœa which obtained so much in London during the prevalence of cholera, and which in a very large proportion of cases ushered that complaint in.

There were 26 cases of small-pox in the Caledonia, while anchored in the Tagus, one of which terminated fatally. The surgeon expresses himself satisfied as to the disease being true variola, but does not give any opinion respecting its origin in the ship, nor any statement as to its being in Lisbon at the time. The first man affected had neither had small-pox, nor cow-pox previously, though an ineffective attempt to vaccinate him was made some time before. In two other persons, one of them an officer nearly 60 years of age, there seems to have been good evidence of their having had the disease in early life, and that in them therefore small-pox occurred twice. Most of the persons affected by the disease had been vaccinated, but the surgeon expresses doubt as to the real vaccine disease having, in all instances, been excited; and gives it as his opinion, that the appearance, manner of progress, and termination of small-pox, in this instance, afford proof that vaccination, though it does not give absolute protection against small-pox, lessens violence, when it does not prevent attack, and confers a large measure of immunity. The subject of the fatal case had been vaccinated, but it does not appear how long before the attack of small-pox.

In a number of concurrent cases of fever, amounting to 20, and designated " synochus," one of which terminated fatally, having the general character of idiopathic fever, there appeared, though not in all of them, a copious pustular eruption—a symptom which the surgeon ascribes to the variolous infection prevalent in the ship, having the power of communicating this feature, and so far modifying the original disease. He expresses some doubt as to whether the fever in question was not a varioloid disease, and therefore dependent on the variolous atmosphere; but the severity of the febrile, and the slightness of the pustular symptoms, seem hostile to that opinion. The question is interesting; and it is remarkable, according to the opinions of the medical officers employed, that in the Tagus, and nearly at the same time, essential fever was greatly modified by the contemporaneous action of other morbific agents; by the cause of cholera in the Asia, aud Donegal, and by that of small-pox in the Caledonia; those other independent agents having the power of communicating to the essential fever some of their peculiar symptoms.

Cholera, of the malignant kind, prevailed. There were 76 cases treated, 19 of which, in the Malabar, were the results of common atmospheric, and dietetic causes, and require no remark. Out of the 57 remaining cases, 17 terminated fatally; they were in the Asia, Donegal, Stag, St. Vincent, Talavera, and Revenge. After some details, we find it stated :—

What the cause of this formidable disease may have been, here and elsewhere, this is not the place to inquire, even if it could be done with the prospect of arriving at a satisfactory conclusion. It may be admitted, however, to observe, that whencesoever derived, and wherever operating, it is essentially one and the same; and that in the case under consideration it was derived from the shore, whence it was carried, through the medium of the atmosphere, to the ships. It may be further stated, that there was no evidence of the disease being propagated by personal contagion.

*Year* 1834.—The ratio of mortality was lower in 1834 than in any of the preceding four years, except the first of the series, 1830; that is calculating all cases of death, whether resulting from external injuries, or disease; for deducting the former, which were less in 1830 than in 1834, the mortality was considerably lower in the latter than in the former.

The near approach to uniformity in the rate of mortality in these commands is striking. In a series of many years it will be found to vary very inconsiderably; the greatest difference between any of the five years examined is three per 1,000 of force, the lowest being 10 per 1,000 in 1830, the highest 13 per 1,000 in 1833. The reason why the difference is so slight is, of course, the rare eruption of destructive epidemics. On such stations as Jamaica, and the West Coast of Africa, the difference between two years, one immediately succeeding the other, is frequently great, the rate of mortality not exceeding that of the Mediterranean in one of them, quadrupling it or more, in the other. Even the outbreaking of a new and highly fatal form of disease, when limited to a section of the force, and not prevailing extensively there, as happened at Lisbon in 1833, does not always much augment mortality, its effects being apparently countervailed by immunity from other, and ordinary causes of death. Thus, in the year just named, the ratio of mortality was higher by a fraction only, than in the two antecedent years, when the cholera in question did not exist; though a fourth part of the total mortality by disease on the station, in that year (1833), was occasioned by it. In the West Indies, and Western Africa, it is often far otherwise, from the operation of endemic fevers, the ordinary, and often evolved products of the places in which they show themselves.

The ratio of the total mortality of the year, including the deaths in home hospitals of patients received from the foreign stations, was 11·1 per 1,000 of the mean number employed, 97 deaths from all causes, in and from the squadrons, having occurred. But 17 of these were in home hospitals; so that there were 80 deaths from all causes, on the foreign stations, being in the ratio of 9·1 per 1,000 mortality of force. Of these 80 deaths abroad, 23 resulted from external injuries, and accidents, reducing the number of deaths, by disease, on the stations, to 57, and giving the very low proportion of 6·6 per 1,000 of force dead by disease during the year, within the limits of the commands.

The mean number of men and officers employed during the year, was 8,745, being an increase of 909 on that of the preceding year. The total number of sick and injured was 11,393, being in the ratio of 1,302·8 per 1,000 of the employed. This ratio is nearly the same as the corresponding one of 1833; the proportion of injuries was, however, larger in the present year than the last, rendering the proportion of disease less, and reducing the ratio of sick to about one in the 100 of the employed.

There were 27 cases of small-pox, three of which terminated fatally. In the Asia there were 18 attacks, and two deaths. She was at anchor in the Tagus during the continuance of the disease, which was nearly four months. The surgeon states that it was generally slight, in some causing scarce any febrile disturbance; and that all those affected had been vaccinated, but at what previous period does not appear. The appearance, progress and power of small-pox in this instance, do not tend to lessen the obscurity which involves the important question—to what extent vaccination has the power of preventing or mitigating, attacks of that disease.

In the Tyne there was one death out of eight attacks. The first case occurred in the person of one of the lieutenants, soon after his return from an excursion to part of Egypt, while the ship was at anchor on the coast. He was not aware of having been exposed to the disease during his absence; he had been vaccinated, and got over the attack easily. In six other cases the disease was not severe; in one it was confluent, and proved fatal. The last case was the only one in the ship, where there was not evidence of previous vaccination, or small-pox. The origin of the disease cannot be traced; but it is satisfactory to know that the fatal case occurred in a person who had not been vaccinated, while others who were more slightly affected, had undergone that operation.

*Year* 1835.—The ratio of total mortality, resulting from service, in the Mediterranean, and on the shores of the contiguous Peninsula, differs in a very slight degree from that of preceding years, taking the deaths from all causes, and whether occurring on the foreign stations, or in the persons of patients sent thence to home hospitals. We shall notice a few facts.

In the Barham there were 34 cases, designated typhus fever. The disease made its appearance soon after anchoring in the Bosphorus, close to the Valley of the Sultan, which is represented as abounding in fever exciting miasmata, and to which the fever in the Barham is ascribed by the surgeon. Up till the period of her anchoring there, she was free from fever. In such circumstances typhus was not to be apprehended, nor is it usual to apply the term to fever derived from such a source; remittent fever, though perhaps without well-marked periods, and with congestive symptoms, was to be apprehended. Two out of the 34 cases proved fatal.

In the Tweed there were 13 cases of fever, designated "mixed," four of which terminated fatally. The ship left Lisbon on the 1st of August, and did not return from the west coast of Africa till the 24th of October, during which time the fever, with its fatal results, broke out and terminated. During her service on the African coast, she anchored a week off the Rio Gambia, for the purpose of procuring wood and water—an employment always attended with danger on such a coast. In this instance the ship's company were not so employed, the work being done by Kroomen, but mere proximity to the shore was sufficient apparently for the production of the disease. A few days after leaving the anchorage, fever made its appearance on board. It did not extend far, but had a severe character, and occasioned a large proportionate loss of life.

Rheumatism was common, and often extremely intractable. It is a curious fact, not generally known, being at variance with commonly received opinions, that rheumatic affections, which obstinately resist treatment in the

Mediterranean, and other places of similar climate, often get rapidly well in England. During the year, 470 cases of the disease were treated ; of which 16 were invalided, being two-thirds of all the invalided of this order of disease, " inflammation with fever."

Altogether from diseases of the lungs, acute and chronic, there were 37 deaths, making a large proportion of the mortality of the year, the total number dead, by disease, being 89.

These and similar facts which have been formerly stated, do not furnish a flattering account of the influence of the Mediterranean in pulmonic disease, either in a preventive, or curative point of view, comparatively, at least, with other naval stations; yet it has been and still is the practice, in recommending a foreign residence to consumptive patients, to give the preference to the shores of that sea.

Under the head of "ulcer," 499 cases were reported; of which 31 were sent to hospital, and seven invalided from ships. Generally the disease was mild and tractable, the ulcerative process having little force, and loss of substance being inconsiderable. The Caledonia again suffered the most, having had 103 cases, seven of which were sent to hospital, and one was invalided. In her there was prevailing tendency to erysipelas, with which the ulcerative action was associated. A similar tendency existed in the ship last year, and during several preceding years; and it is therefore probable, to whatever common causes the crew might be exposed, that there was some peculiar agency in the ship herself causing that tendency.

*Year* 1836.—The ratio of mortality, in the joint squadrons, in the six successive years which have been enumerated, was low, the highest, that of 1833, being no more than 13 per 1000 of the active force, the result of disease and injuries, not only on the stations, but in home hospitals, in patients received from the Mediterranean, and Peninsular commands. During the year 1836, the ratio of mortality, from the same sources, and in the same places, was as low as 7·9 per 1,000 of the mean number employed, the total number dying on, and from the station being 89, viz. 76 abroad, and 13 at home. But of the total 89 deaths, 16 were from external injuries, reducing the mortality by disease to 73, which is in the ratio of 6·5 per 1,000 of mean strength. The rate of mortality, from all causes, on the stations, accidents as well as disease, was 6·7 per 1,000 of force, and from the latter, little more than 5 per 1000. The results, whatever view may be taken, are highly satisfactory, the entire loss to the service being so small; if the mortality, from disease on the stations be looked at, separate from death by external injuries, and in the persons of invalids at home, it will appear strikingly low, only one death, to every 188 men employed, having occurred.

It has been observed that, during the six preceding years, the annual rates of mortality had differed very inconsiderably ; and it was stated that, in such positions, rarely visited by severe epidemics, a pretty close approach to uniformity might be expected. This year forms an exception, difficult to account for; because, in that respect—immunity from any destructive epidemic—it had little advantage compared with the six preceding years. It is true that malignant cholera affected two, which, on account of a return being mislaid, was extended to three of them; but during the two last, 1834 and 1835, only five deaths resulted from the disease, two in the former and three in the latter; to account for different rates of mortality, these numbers would avail little. Even in 1833, when there were 17 deaths from cholera, the ratio of mortality scarcely rose above that of other years, when no such disease existed.

The causes of such difference, in such circumstances, were no doubt traceable, could all the ordinary agents called non-naturals, injuriously applied, producing, and aggravating disease, be detected, and duly appreciated; as they cannot, the necessity arises of considering it accidental. One of the sources of difference between the year in question, and the immediately preceding year, was the small proportionate loss from acute diseases of the lungs in this, compared with the former. The agencies producing that source were certainly not unimportant, nor accidental, as to their operation; but why their operation was so much more powerful in the first, than in the last, does not appear. Another and principal source of difference was the much higher proportionate loss from external injuries in 1835, than in 1836; this was however accidental, in the proper sense of the word.

But though the rate of mortality was much lower in the present year, than in the six preceding years, the rate of sickness was not. It was indeed higher than in the most of them; it was higher considerably than in the immediately preceding one, the rate of mortality being lower by a third. The total number of sick and hurt during the year was 14,623, being in the ratio of 1292·3 per 1,000 of mean strength. The ratio per 1,000 of force, of cases sent to hospital, was 44·4, differing but little from any of the preceding years, except 1831, when it was 71·9. The ratio of invalided, like that of dead, though not in equal proportion, was the lowest of any of the seven years. It was, including the invalided in home hospitals, and on all accounts, chronic disease, debility, age, and effects of injuries, 21·6 per 1,000 of strength; so that the total loss to the squadrons, by invaliding, added to death, was under three in every hundred of the mean number employed. It does not follow that the loss to the service was so much; for a number of the invalided, on the foreign stations, recovered health and strength at home, and would sooner or later return to it.

There were 36 cases of small-pox in the Hastings, at Lisbon, two of which terminated fatally. The surgeon's report, as to the powerfully modifying influence of vaccination, in this instance, is satisfactory. In one of the fatal cases, the subject had not been vaccinated; in the other, though the operation had been performed, the cicatrix was indistinct; in a severe, confluent case, there was no mark of vaccination; while in all the instances, in which the scars were considered satisfactorily distinctive of effective vaccination, the disease (small-pox) was remarkably mild.

### GENERAL RESULTS.

The Reporter passes to a general review of the condition of the naval force in the Mediterranean during the last seven years. He first presents a Table showing the total number of cases; the number of all diseases and injuries, in classes; the number of cases sent to hospital, invalided and dead; with the ratio of each per 1,000 of mean strength.

"These figures," says he, "furnish a gratifying account of the health of the naval force, in the Mediterranean, and on the shores of the adjacent peninsula, during the seven years to which they refer. They would no doubt be more satisfactory, if they embraced a longer period, as they ought, were it the object of the Report to exhibit, not only the state of health now enjoyed, but also to compare it closely with what it was in former, distant times, and to show its progressive improvement. In order to obtain the latter objects, it would be necessary to go back at least fifty or sixty years, when, however, and indeed long afterwards, no documents were furnished, from which any thing like correct conclusions could be drawn. The total number dying might be pretty nearly ascertained, but the separate causes of death, at sea, and the sources of those causes, when traceable, must be mere guess-work. Were it possible to institute an accurate comparison between the health of the Navy, as it then was, and now is, the difference, in favour of the present condition would be found immense, and render it more striking even than it is. As to the rate of improvement through a long series of years, it would not be found at all steadily progressive; it would rather be found to have made great advances at certain well-marked periods. One, and the most remarkable, of the advances in improvement took place about the close of the last century, and soon after the time, when, among other advantages conceded to the fleet, the system of victualling was placed on a fixed, and sufficient footing; when the nominal became the real ration of the sailor, and the fraud and iniquity perpetrated under the cover of *pursers' weights* were abolished. From that period, till the termination of war in 1815, the progress of improvement was not rapid. The health condition varied at different times and places, in connexion with the nature, and localities of service, but no great change, pervading the whole, can be traced. Peace, however, made many beneficial changes in the condition of the naval force, and with them came increase of health. Service was altogether voluntary, of short and defined duration, and free from many restrictions, and privations, which were necessary, at least in reference to impressed men, during war. Meat could be issued soon after it was salted, and biscuit after it was baked; and they were consequently more palateable, and nutritious, than they had been, though the same in quantity, in the latter years of the war. These, and other changes, including the power of selecting seamen, the diminution of the allowance of spirits, and the issue of tea, or coffee, instead, have raised health to its present high standard."

The average annual rate of mortality, during these seven years, was 11·1 per 1,000 of active force, the total number dying, on the stations, and from them, being 617, the aggregate number employed, 55,709. Of the 617 fatal cases, 101 were from external violence, and 97 occurred in patients from the stations at home; so that no more than 419 deaths resulted from disease abroad. Deducting the deaths from external causes, the mortality by disease on the stations, and from them, in home hospitals, averaged 9·3 per 1,000 per annum; and taking the deaths on the stations, resulting from disease, and independent of violence, the annual rate of mortality, was little more than 7 per 1,000 of the employed.

Low, however, as the rate of mortality is in these commands, it is higher than in South America, where the average of the same seven years has been shown to be no more than 7·7 per 1,000 annually of active force, the effects of disease both on the station, and in home hospitals, from it, being on the station 6·5 per 1,000. Compared with the West Indian and North American command, the mortality in these commands, the effects of disease, is almost exactly half.

The average number of sick and hurt was 1304·6 per 1,000 per annum of force; of sick, separate from hurt, 1081·7 per 1,000. These averages differ very little from the corresponding ones in South America, where the sick and hurt averaged 1310·7, and the sick alone 1071·8 per 1,000, per annum of force; but it will be seen that the proportion of injuries was smallest in the Mediterranean, the total number treated being rather less, while that of sick was more than in South America.

The total loss sustained by the squadrons in the Mediterranean, and on the neighbouring coasts, from the number invalided, added to the number dead, averaged 36·9 per 1,000 of force annually, the mean annual rate of mortality being, from all causes, abroad and at home, 11·1, that of invaliding, in the same terms, 25·8 per 1,000 of the number employed. It is remarkable that the entire loss sustained by the South American squadron, during the same time, was precisely the same, the larger proportion of invaliding there being exactly balanced by the greater mortality here; the average yearly mortality of the South American squadron was 8·9, of invaliding 28 per 1,000 of the employed.

The next Table shews the total number of cases; the number of all diseases and injuries, in classes; the number invalided and dead; with the ratio of each per 1,000 of attacked.

The loss sustained by the squadrons during these seven years, from all forms of essential fever, was slight, the average mortality being under two per 1,000 annually of the employed. It was more, however, than in South America, where the mortality averaged only 1·3 per 1,000 per annum of the employed. In these commands, though considerably greater than in South America, it is small in itself, and insignificant, when compared with the West Indies. There, though the ratio was much lowered, it may be assumed by a third at least, on account of the West Indian force being mixed up with the North American, the average annual mortality was 11·2 per 1,000 of the employed; it was therefore about six times as much as in the Mediterranean, nine times as much as in South America. But though the proportion of dead was higher in the Mediterranean than in South America, that of affected was lower; the annual average of cases was 84 per 1,000 of force in the former, while it was 115 in the latter. In both instances, especially the latter, the disease, in a great majority of cases, had little force, and was frequently, there is little question, slight symptomatic, rather than primary, fever. The remark, though less generally, may be applied to disease designated fever in the West Indies; for while 264 died, 4,932 were treated there under that name.

The affections classed under the head of "organic diseases of the brain" amounted to 113 cases in seven years; of which 20 were sent to hospital, 12 were invalided, and 42 terminated fatally. They include acute inflammation, palsy, and apoplexy, but are composed chiefly of the two latter, which furnish all but one of the fatal, and all the invalided cases. The ratio of attacked, as well as of dead, is much higher than in South America, or the West Indies.

There were 1,742 cases of inflammation of the lungs, and their membranes and air-passages, exclusive of catarrh, and influenza; of which 249 were sent to hospital, 34 were invalided, and 54 terminated fatally. The annual rate of attacked averaged 31·3, of invalided 6, of dead 1, per 1,000 of force. In comparing these with the corresponding ratios in South

America, and the West Indies, it appears that they are all unfavourable to the Mediterranean, except that of invalided, in South America, for which want of hospitals there, independently of diseases' force, will satisfactorily account. Thus, in South America the average annual rate of attacked was 28, in the West Indies 22, per 1,000 of force; while the annual ratio of mortality was, in the former, 1 in 2,400, in the latter, 1 in 1,100, of the employed. The mortality from these diseases is strikingly low in South America.

Of all forms of hepatic disease, designated inflammatory, there were 403 cases; of which 62 were sent to hospital, 25 were invalided, and 12 terminated fatally. The annual ratio of the first is 7·2, of the second 1·1, of the third ·5, of the last ·2, per 1,000 of the employed. The infrequency of such disease in the Mediterranean is remarkable.

The loss by primary inflammation of the alimentary organs, including the stomach and bowels, with their membranes, was very slight.

But, while these affections of the alimentary organs were less frequent and fatal, than in the other two commands, organic diseases of the pulmonic apparatus were much more frequent than in either, and, including primary inflammation, more fatal, especially when compared with South America. In the Mediterranean 285 cases were treated under the name of "phthisis;" of which 127 were sent to hospital, 70 were invalided, and 105 terminated fatally. These fatal terminations include the deaths not only on the stations, but in home hospitals, from them, the total resulting mortality, in fact; the same remark applies to all diseases in the seven years' summary; it may therefore be inferred that the term phthisis was not, in every one of the 285 cases, correctly applied. The average ratio of such cases was 5·1 per 1,000 per annum of force, while in South America it was 3·2, and in the West Indies 4·8. The annual ratio of invalided was 1·3 per 1,000 of force, while in South America it was little more than half; in the West Indies it was 2·4: why it was comparatively high there does not appear. The average ratio of dead was 1·9 per annum, being the same as in the West Indies, while in South America it was 1·5 per 1,000 of the employed.

In the year 1831 there were nine deaths from erysipelas, which have been placed in the Table of principal diseases, though, from the infrequency of the affection in a severe form, in the other commands it was omitted. During the other six years in this command, other fatal cases occurred, but not having a common source, and not appearing to possess essentially the same character, they are noticed among diseases which do not prove frequently fatal to seamen.

There were 742 cases of dysentery, of which 88 were sent to hospital, 16 were invalided, and 18 terminated in death; the ratio of attacked was 13·3 per 1,000 of invalided, and of dead 1 in 3,000 each per 1,000 annually of force. The ratio of attacked was a little more, of invalided half, and of dead the same, as in the West Indies. Comparing them with South America, that of attacked was little more than half, of invalided, and dead, not a third.

Venereal disease was frequent, no fewer than 4,222 cases of it having been treated, 2,771 of which were syphilitic, and 1,451 gonorrhœal. The average ratio of the first was 49·9, of the last 26 per 1,000 annually of force; the temporary reduction of force by these forms of disease was therefore considerable. Compared with the West Indies, they were more numerous by a half, with South America by a third. But they were all, though so numerous, cured on the station, whereas 15 were invalided from the West Indies, and 13 from South America.

Rheumatism was not so prevalent as in the other two commands. The annual average ratio of attacks was 63·9 per 1,000 of force; in South America it was 72·3, in the West Indies and North America 69 per 1,000 annually of force.

But while rheumatism was less frequent than in the other two commands, and less severe than in the West Indian, especially as measured by the ratio of invaliding, the proportion of catarrhal cases was considerably greater here than in the West Indies and North America, much greater than in South America. Here the average ratio of attacks was 201·7 per 1,000 annually of force: in the West Indies and North America it was 181·8, and in South America 137·8 per 1,000 annually of force. Out of the total number of cases, which in seven years amounted to 11,237, 12 terminated fatally; three cases in the West Indies, and one case in South America terminated fatally. The two diseases, rheumatism and catarrh, are attributed to similar atmospheric agency, in which the most notable property is reduction of temperature; and it is believed that the excitation of one, or other depends chiefly on the condition of the subject, as to degrees of susceptibility existing in different textures of the body. The opinion has prevailed during all ages, and is no doubt generally just; but it is no less true that there are often other, less evident, and little suspected, agents in operation, especially in the production of disease designated catarrh. In the cases under consideration, it appears, that while catarrh was much more prevalent in the Mediterranean than either in the West Indies and North America, or South America, especially the latter, rheumatism was less prevalent and much less detrimental, particularly in comparison with the West Indies and North America.

### INFLUENCE OF FORMS OF SHIPS ON HEALTH.

"As vague and conflicting opinions are entertained in the service respecting the comparative salubrity of different classes of ships, and as the subject is one of intrinsic interest, it is desirable to submit it to the test of numbers, so far as the peace constitution of squadrons will admit of its being settled. The question can only be tried in such commands as have a considerable number of ships of all classes: that was the case in no other than the Mediterranean, and on the Peninsular coasts, and to those the inquiry is necessarily confined. It was an object to make a distinct class of steam vessels, and their number being very small, till the year 1834, the comparison begins with that year, and is carried on through the two following, so that it embraces the total force of the joint squadrons, during three years, the aggregate numbers employed being 28.908."

Dr. Wilson admits that the time and the numbers are yet too limited for absolutely settling the question. Still an approximation to the truth is attainable.

For the object in view, all the ships and vessels employed have been divided into four classes.

The first class embraces all ships of the line, whether they have two, or three gun-decks; the second all frigate-built ships, whatever their tonnage, or weight of metal may be; the third all flush-decked vessels, sloops, brigs, schooners; they will be referred to as a class, under the general name of corvettes; and the last vessels of which steam is the principal propelling power.

There is much difference in the size of the vessels composing each of these classes, on the sanatory power of which, in connexion with the numerical force of their respective crews, it is not intended to enter here; but it may be stated, that the complements of ships of war are pretty closely adjusted to their sizes, to whatever class they may belong; and further, that difference in size, in the same class, does not appear to possess much influence on health. But in each of the classes, whatever be the size of the individual vessels, there is a peculiar form, giving a particular distinctive position to the places where the crews sleep, and eat, which may be presumed to act on health; at least, this is true—the particular position of the inhabited parts—of all the classes, except the two last, in which there are other circumstances which differ greatly, which are likely to have much influence, the power of which it is desirable to determine.

In the first class, composing ships of the line, the part of the ship occupied by the crew, at meals, and during sleep, has ports, and at least one deck between it and the holds, store-rooms, and wells; there is also a gun-deck over it, intermediate to it, and the upper deck. In frigate-built ships of whatever size, the inhabited deck is immediately over the holds, &c.; over it is the gun, or main-deck, and over that the upper or quarter, and forecastle deck. The inhabited deck has no ports, and is deprived of those means of ventilation. Scuttles are cut in the sides for that purpose; but they are so close to the water, that they are available only in fine weather, at anchor. Corvettes, forming the third class, have no gun-deck, in the language of the service. Their guns are planted on the upper, flush, or weather deck, between which and the holds there is but a single deck, without ports for the accommodation of the crew. In general form, the fourth class, that of steamers, is like the last, the inhabited part having the same relation to the holds, and the open air; but they have the addition of the steam apparatus, and, when at sea, augmented temperature, and evolution of steam.

Four Tables are given for the purpose of exhibiting the kind and the frequency of disease that obtained in vessels of seventy-two guns and upwards —in frigates—in corvettes—and in steamers.

The largest proportion of mortality resulted from service in frigates, the next in corvettes, the next in ships of the line, the smallest being in steamers. In the first, the annual rate of mortality on the average of three years, was 9, in the second 8·1, in the third 7·6, in the fourth 5·4, per 1,000 of the mean number employed. The ratios are deduced not only from deaths occurring on board, but also in hospitals abroad and at home, of patients sent from all the vessels composing the four classes. To arrive at correct conclusions, it was necessary to ascertain the results of disease, originating in each of the classes, in hospital; because there is great difference in the number of patients sent thither, apart from the frequency and violence of disease, the difference depending partly on the will of commanding, and medical officers, but principally on the contingency of being at sea, or in harbours having hospitals. This is strongly exemplified by the relative numbers sent to hospital from the second, and fourth class—from frigates, and from steamers ; being from the last, in a ratio three times as high, as in the first. Steamers, being almost always employed in making passages, and making them quickly, have frequent intercourse with hospital harbours, which, with inferior accommodation for sick on board, accounts for the large proportion thus disposed of. How far the frequent opportunities they have of sending their sick speedily to hospitals, may contribute to lower their ratio of mortality, cannot be determined; because it cannot be known to what extent the results of disease would have differed, if all the sick, or a great proportion of them, had been kept on board. But it may be presumed, considering the apparent advantages afforded by hospitals, that it would have been augmented in that case, in some degree, whatever the degree might have been. It will be seen, while their ratio of mortality was so low, their ratio of sick was much higher than that of frigates; it was the highest indeed of all the classes, except that of corvettes, than which it was very little lower. This, however, is a very uncertain measure of the force, and fatal tendency of disease.

The ratio of invalided did not, from the nature of things, differ so much as that of sent to hospital ; yet the difference between all was considerable ; between two, the first and last, great. In the first, the average ratio was 22·1, in the second 17·5, in the third 20·1, in the fourth 13, per 1,000 per annum of force. The remark as to the ratio of dead, applies equally to this ; namely, that it is deduced from the total number invalided, whether on board, in foreign, or in home hospitals, from each of the classes. And it appears, that the two classes which had respectively the highest and lowest proportion of deaths—the frigates and steamers—had the lowest proportion of invaliding, though it was considerably less in the steamers, which had the lowest, than in the frigates, which had the highest mortality ; while in ships of the line, which, after steamers, had the lowest proportion of deaths, the rate of invaliding was the highest, nearly doubling that of steamers. Part of the difference in these results—the want of proportion between the number sick, sent to hospital, invalided, and dead, though the second should scarce be enumerated—may be the effect of contingent, not permanently operating causes in the respective classes; and it probably is so.

*Essential Fever.*—The ratio of attacks did not differ materially in the different classes, the lowest being 65·4, the highest 76·8 per 1,000 annually of force ; the first was in ships of the line, the last in frigates. But there was great difference in the rates of mortality, being double in one, and more than double in another, what it was in the other two. Thus, it was 1·1 in ships of the line and steamers, while it was 2·2 in frigates, and 2·7 in corvettes, per 1,000 annually of force. As respects attacks, the steamers had nearly as large a proportion as frigates, in which the mortality was double, and a larger proportion than corvettes, in which the mortality was more than double.

On the other hand, the steamer class suffered more from primary inflammation than any of the other classes. By death, their loss was in the ratio of 2·2 per 1,000 annually of force; in ships of the line and frigates, it was 1·8 each; and in corvettes, it was 1·2. By invaliding, the loss of the steamers was 8·7, while in ships of the line it was 4·9, in frigates 6·4, and in corvettes 6·8, per 1,000 annually of force. The high heat alternating rapidly with comparatively severe cold, to which the crews of steam ships, particularly the engineers, are exposed, was probably the cause of, or, at least, would appear to account for, the greater force, and more intractable nature of inflammatory affections in this class. They were also more frequent in it, than in any of the other classes, except the third, in which the mortality from this class of disease was the lowest.

"In the hæmorrhagic class of diseases, which comprises phthisis and hæmoptysis, the discrepancy is surprising, and so great as to be irreconcilable with all that is known of those diseases. The frequency of recovery from phthisis, in the service, or, in other words, the comparatively low proportion of deaths to attacks, has more than once been adverted to; and it has been stated, that part, at least, of such apparently favourably results, might arise from occasional error in diagnosis, the term 'phthisis' being applied to affections which did not possess its real nature. This, looking at the high ratio of cured, in connexion with the obscurity of the primary symptoms, and the difficulty often encountered, in ascertaining the distinctive physical signs, at sea, may be safely asserted, without imputing want of care or intelligence to the medical officers. But though generally applicable to a certain extent, it cannot be supposed to operate more in one class of ships than in another; and the discrepancies between the various classes, in this respect, in this disease—the number attacked, and the ratio dead—appears unaccountable.

It is remarkable that the proportion of attacks increased, though not in a regular ratio, in the inverse order of the size of the different classes, so far as they are distinguished by size, Thus, in ships of the line it was 9.1, in frigates 10.6, in corvettes 18.6, in steamers 23.9 per 1,000 annually of force. The same remark applies to the numbers sent to hospital; the smallest proportion was from ships of the line, rising through the others, and being much greatest in steamers. It does not apply to invalided, the annual ratio being the same, viz. 3.3 per 1,000 of force from liners and corvettes, 2.6 from frigates, none being invalided from steamers. Comparing the number dead with the number attacked, and sent to hospital, in the various classes, with relation to the mean number employed, the order of things was reversed, the proportion of mortality being highest in the large, lowest in the small. Thus, in ships of the line it was annually 2.4, in frigates 2, in corvettes 1, in steamers 1.1, per 1,000 of force. It was low in them all, strikingly so in the two last, and rendered more striking in steamers, by the large proportion of cases reported in them. About two-thirds of all the cases comprised in this order of disease, the hæmorrhagic, are placed under the head of phthisis. The number in steamers amounted to 22; supposing, not two-thirds, but the half of them, to have been phthisical, how is the very small resulting loss, viz. one death out of the whole number, to be accounted for?—Does steam act curatively in such cases?"

### II. Births, Deaths, and Marriages in England.

We turn from the navy to the Second Annual Report of the Registrar General for England. A document of no mean consequence and merit. For much of it is devoted directly and specifically to medical statistics and to the improvement of medical science. The attention of the government of this country is beginning to be directed to this object, and it requires no gift of prophecy to foretell that both the legislature and the executive must extend more assistance to it, than has heretofore been given.

The Registrar compares the results of the last year's registration with those of the preceding one.

The numbers registered in the year ending June 30, 1839, were—

| | |
|---|---|
| Births .......... | 480,540 |
| Deaths .......... | 331,007 |
| Marriages ........ | 121,083 |

which, compared with the numbers for the preceding year, shew, for births an increase of 80,828; for deaths, a decrease of 4,949; for Marriages, an increase of 9,602.

The increase of the number of registered births is attributable to the success of the plan of registration. A registration of births has thus been effected for the second year of registration, approaching much nearer to a complete record of the whole number born, than was afforded by the registers of baptisms for the 10 years ending 1830, the latest period at which we possess authentic returns with respect to such registers. The mean annual number of registered baptisms during that period was 375,349; and if it be assumed that the number of such registered baptisms has increased in the same ratio in which the population increased from 1820 to 1830, it will appear that the probable number registered in the parochial registers for the year ending June 30, 1839, will not have exceeded 460,000, a number less than that of the number of registered births for the same period by more than 20,000.

The Registrar ascribes the decrease of registered deaths to a really diminished mortality. He believes that that of the preceding year was above the average, owing to the inclemency of the winter in the beginning of the year 1838, and to some epidemics, the prevalence and severity of which appear to have subsequently declined.

The Registrar thinks that it is impossible, at present, to do more than approximate to a solution of the important question—What is the proportion of the mortality to the population of England and Wales? The only data for arriving at an estimate of the population for the year ending June 1839, are the returns of 1821 and 1831. Taking the enumerations then given,—adding the Scilly Isles, which were omitted in 1821,—adding for the army, navy, and other unenumerated population, according to the statements prefixed to those returns, such proportions as may be assumed to have belonged at these periods respectively to England and Wales, the numbers will be as follows:—

| | |
|---|---|
| 1821 ........ | 12,162,056 |
| 1831 ........ | 14,055,562 |

These numbers include all who may be assumed to have incurred the risk of death within England and Wales at those periods, and whose deaths might have been registered, if an act similar to the present had then been in operation, all of whom were not included in adverting to those returns in my report of last year.

With these corrections, and on the assumption that the rate of increase since 1831 has been the same as from 1821 to 1831, the population of whom the deaths might have been registered, may be estimated to have been nearly as follows, at the middle of each of the two first years of registration under the present law:—

| | Males. | Females. | Total. |
|---|---|---|---|
| January 1, 1838 .... | 7,612,967 | 7,828,768 | 15,441,735 |
| January 1, 1839 .... | 7,723,924 | 7,942,876 | 15,666,800 |

The deaths registered in the years of which the above-mentioned periods are the middle terms, were—

| | Males. | Females. | Total. |
|---|---|---|---|
| Year ending June 30, 1838 .. | 170,965 | 164,991 | 335,956 |
| Year ending June 30, 1839 .. | 169,112 | 161,895 | 331,007 |

Without correction for omissions, this would shew the mortality to have been as follows .—

| | Males. | Females. | Total. |
|---|---|---|---|
| 1837-38 ...... | 1 in 44·5 | 1 in 47·5 | 1 in 46· |
| 1838-39 ...... | 1 in 45·7 | 1 in 49· | 1 in 47·3 |
| Mean of the 2 years | 1 in 45·1 | 1 in 48·2 | 1 in 46·6 |

Assuming that the population may be estimated as above, and that it is unnecessary to allow a greater correction than 2 per cent. for omissions in the registration of deaths, the mean mortality of the two sexes for those two years will have been about 1 in 46.

With the marriages of the people, or with their education, points discussed by the Registrar, we have no immediate business ; nor need we therefore go into them. We may simply mention that, in 15 English counties, and in North and South Wales, more than 40 per cent. of the men were unable to write their names ; and in 19 English counties in the West Riding of Yorkshire, and in Wales, more than half the women were similarly deficient. It deserves mention also, that there is a decided superiority with regard to education in the metropolis, as compared with the rest of England and Wales, and, next to the metropolis in the north of England; and that the principal deficiency is in Lancashire, Bedfordshire, Monmouthshire, and Wales.

In 4,858 marriages, the average age of marriage was, for men, about 27 years, for women, 25 years and a few months.

The Registrar has made some essential alterations in his plan.

" In exhibiting," says he, " abstracts of the number of deaths at different ages, for the year ending June 1839, I have adopted an arrangement different from that which was employed for the first year of registration ending June 30, 1838. Instead of exhibiting enumerations of deaths at every successive year of age, I have divided the periods at which death occurs in a manner more conducive to the attainment of those purposes for which such enumerations are made. I have divided the deaths in the first year into six periods ; during the following four years, I have shewn them for each separate year ; and, after that age, for quinquennial periods. I will briefly state the reasons for this change.

With respect to the subdivision of the first year, it must be observed that more than a fifth of the whole number of deaths registered in the year ending

June 30, 1838, namely, 71,888 out of 335,956, are under one year of age; that the distinction of months at that early period will exhibit circumstances more important with respect to the expectation of life, than that of years at later periods; and that the expectation of life on the day of birth differs greatly from that of six, three, or even one month old. It appeared to me, therefore, that such distinctions ought not to be overlooked; and that the abstract should be framed rather with reference to the ascertained ratios of mortality, than to an equal division of the periods of age. After the first year, the ratio of mortality rapidly declines; and this decrease is shewn by the enumeration of deaths for each of the four following years."

"After the fifth year," he goes on to observe, "I have combined the ages in quinquennial periods, a system which, after much consideration, I deemed preferable to that adopted in the abstracts for the first year of registration, namely, of stating the number of deaths at each successive year of age.

To the statement of deaths at each successive year, it might be objected that it was delusive, and assumed an appearance of minute accuracy which was not founded on truth. This objection is not applicable to the reported ages of children. Their recent births are fresh in the recollection of their parents or guardians, and their age is stated with sufficient accuracy. But it is not so with respect to the ages of persons far advanced in life; many of whom, especially among the poorer classes, are ignorant of their exact age, and when they die, leave no record which enables their surviving relatives to state their ages with precision. An evidence of the vagueness attending statements of age is 'the tendency to speak in round numbers' noticed in the preface to the abstract of the population for 1831, a tendency causing a great apparent excess of mortality in the decennary periods at 30 and upwards, and of which the following remarkable instances may be found in the abstract of ages published in the preface to the population abstract for 1831, extracted from Burial Registers in England and Wales for 18 years :—

| Ages. | | Ages. | | Ages. | | Ages. | | Ages. | |
|---|---|---|---|---|---|---|---|---|---|
| 29 | 26,630 | 39 | 23,778 | 49 | 23,689 | 59 | 25,782 | 69 | 33,038 |
| 30 | 32,027 | 40 | 33,513 | 50 | 33,527 | 60 | 43,273 | 70 | 53,953 |
| 31 | 22,301 | 41 | 20,989 | 51 | 20,911 | 61 | 26,084 | 71 | 32,162 |

Experience has shewn that this incorrectness also exists in the statements of ages in the registration of deaths, as will appear upon reference to the abstracts for the year ending June 30, 1838.

An abstract of deaths at every successive year of age is, therefore, confessedly incorrect; and, in stating this, I am stating a strong reason against its continuance; for by exhibiting such an abstract, I should commit a fault which I deem it most important to avoid,—that of assuming the delusive appearance of more minute accuracy than actually exists. By combining the deaths at different ages, after the fifth year, in quinquennial divisions, not only are errors and irregularities materially diminished, but the abstracts are rendered in a form more useful, more conducive to the fulfilment of those practical objects for which such abstracts are principally compiled. The most important use of abstracts of deaths is their application to the construction of tables of mortality; which, it must be remembered, are constructed, not from enumerations of deaths alone, but from two series of facts,—the numbers living at different ages, and the numbers dying at the same ages, and the observed relation between those facts. This relation of the living to the dying is varying daily. But it is obvious that however complete might be the record of facts, complete beyond all conceivable possibility of attainment, these variations in the minuter portions of time would be too irregular for the safe deduction of any general laws; and that it is only by including large numbers of facts, and long portions of time, that we surmount the difficulties which such casual irregularities create, and arrive at the ascertainment of any well-founded laws of mortality.

In the assignment of these periods, the quinquennial division is found to be recommended, both by its correspondence with the enumeration we already possess of the ages of the living, and by the authority of those who have already adopted it. The ages of the living in 1821 were enumerated for quinquennial periods up to the age of 20, and for decennial periods after that age. The numbers of the living at different ages were not enumerated in 1831. It is earnestly to be wished that such enumeration may be made in future, and for quinquennial periods beyond the age of 20; but it is needless to expect that an enumeration more minute than for quinquennial periods, for all above childhood, can be effected with success. If, therefore, the utmost to be expected with respect to the future enumeration of the living is, that it be given for quinquennial periods, it becomes advisable that the age at which persons have died should be given in a corresponding manner."

The extract is important, and we invite attention to the suggestions it contains.

The Registrar remarks that the Carlisle table, the Swedish table, the Northampton table, the Montpelier table, and Deparcieux's table of annuitants were all calculated upon these principles.

The Registrar proceeds to notice the most remarkable diversities exhibited by the Abstracts of the second year of Registration, with respect to different portions of the kingdom.

The most marked and serious difference is that which is observable between the mortality of rural districts and of large towns, as exemplified in the proportion of the deaths of children, and of persons dying at advanced ages. The mortality of children appears to have been greatest in towns; and among those towns respecting which he can exhibit separate returns, the greatest at Manchester, where it appears that out of every 1,000 deaths of males, 496 were of children under three years of age. The mean deaths of children under three years, in Manchester and Salford and suburbs, were 475 out of 1,000 deaths. In Leeds and its suburbs, the proportion was 447; in Birmingham 440; in Liverpool and West Derby, 437; while in Dorsetshire and Wiltshire, it was 281; in Devonshire, 296; in the North Riding of Yorkshire, with Durham (except the mining parts,) and the northern part of the West Riding, 282; and in the northern part of Lancashire, Westmorland, Cumberland, and Northumberland (except the mining portion of the latter,) not more than 253. In the whole of England and Wales, the mean mortality under three years was 343 out of 1,000 deaths at all ages; and it is to be remarked that, notwithstanding the comparative unhealthiness of towns, the proportion in the metropolis is still less, namely, 338.

Equally remarkable are the contrasts exhibited by the towns and rural districts, with respect to the proportion of persons who appear to have died in old age. The proportion out of every 1,000 deaths, which have been at the age of 70 and upwards, has been in Manchester, only 53; in Liverpool, 60; in Leeds, 68; in Birmingham, 78; in the Metropolis, 99; while in the North Riding of Yorkshire, and the agricultural parts of Durham, it is 202: in Devonshire, 208; and in the North of Lancashire, Westmorland, Cumberland, and Northumberland, not less than 210. In the whole of England and Wales, the proportion, out of 1,000 deaths occurring at 70 and upwards, was 140.

Great also are the differences exhibited by the mining districts and the agricultural districts which surround them, with respect to mortality, both in childhood and in advanced age.

In the mining parts of Staffordshire and Shropshire, the mean deaths in 1,000 at all ages under three years, were 462; at 70 and upwards, only 90. In the rest of Staffordshire, Shropshire, and Cheshire, the proportion under three years was 332; at 70 and upwards, 141. In the mining parts of Northumberland and Durham, the proportion of deaths under three years of age was 349; of deaths at 70 and upwards, 150; while in the surrounding agricultural districts comprised in divisions 22 and 24, the proportions of deaths under three were only 282 and 252; and of deaths at 70 and upwards, 202 and 210.

In Manchester and Birmingham, the year ending June 1839 shews an increased mortality among children as compared with the preceding year; but in Manchester there appears to be a diminished mortality between the ages of 20 and 40, and between 55 and 75; in Liverpool, a diminished mortality between 30 and 50; and in Leeds, a diminished mortality between 25 and 50.

But the Abstracts of the first and second years of Registration shew very little difference either in the Metropolis, or in the whole of England and Wales.

We pass now to the Appendix, which consists of a Letter to the Registrar-General from Mr. William Farr. The zeal, the talents, and the accuracy of this gentleman need no eulogy from us, and we accordingly proceed at once to the facts which he has collected and communicates.

The Appendix contains:—

Mortality and Diseases of the Year 1838—Diseases of Males and Females—Diseases of Towns and of the Open Country—Influence of Climate—Influence of the Seasons—Progress of Epidemics—Statistical Nosology—Table of Mortality for the Metropolis, 1840, (reprinted from the Weekly Tables published at the General Register Office.)

Mr. Farr makes some remarks on the utility of Registration, in which we cordially concur. We proceed to—

### Mortality and Diseases of the Year 1838.

The rate of mortality in 1838 was higher than in the latter half of the year 1837. Small-pox and typhus prevailed epidemically. The epidemics began in 1837, close upon the decline of influenza, and attained their acme early in the year 1838. The class of pulmonary diseases was much more fatal, so were the convulsions of young children, and the equally obscure maladies of old age. The rigorous weather, which set in early in January, exercised a decided influence upon several of these diseases; and was, with the epidemics, apparently the cause of the increased mortality.

The annual rate of mortality was:—

| Males. | Females. | Mean of the Two Sexes. |
|---|---|---|
| 2·28 | 2·12 | 2·20 per cent. |
| or 1 in 44 | 1 in 47 | 1 in 45. |

### Diseases of Males and Females.

The mortality of males was seven per cent. higher than the mortality of females; and it is well established that the mean duration of life in females is longer than in males. The Tables exhibit the principal diseases which lead to this result; and show that, while the two sexes are concurrently exposed to the ravages of nearly all the causes of death, their degree of liability to death from particular maladies is very various. The discrepancies may be ascribed to two sets of causes; a difference of organization, and a dissimilarity of habits and occupations, involving different degrees of exposure to the accidents, hardships, and dangers of life. Deaths from childbirth and deaths from violence are examples; 2,811 women died in childbirth, while 8,359 males and only 3,368 females died violent deaths. The higher mortality, and the smaller number of males living, have been ascribed exclusively to intemperance, wars, excessive fatigue, and other external causes; but this ground is too narrow; for the differential mortality is greater in early childhood and before birth than in the more advanced ages, and one of its causes must be sought in the intimate structure and properties of the body.

34,321 males and 33,556 females died of the *epidemic class* of diseases; small-pox, measles, croup, thrush, diarrhœa, dysentery, cholera, and influenza, proved most fatal to males; hooping-cough to females. Typhus, scarlatina, and erysipelas, were scarcely more fatal to males than to females.

The annual mortality from the first class of diseases was 4·5 in 1,000 living; and half the amount, or 2·3 in 1,000, was occasioned by *small-pox* (1·0), and *typhus* (1·3); the former disease, as well as hooping-cough, having been more fatal than in 1837, and the latter nearly the same; while measles, thrush, diarrhœa, dysentery, cholera, and ague had declined.

Diseases of the *nervous system* destroyed 49,704 persons; 26,047 by *convulsions*, 7,672 by *hydrocephalus* (water in the head,) and 2,178 by *cephalitis* (inflammation of the brain,) three common diseases of children.

Mr. Farr comments on the vagueness of the heads—Convulsions—Fits—Teething.

The diseases of the nervous system are 23 per cent. more fatal to males than females, the chief difference arising from the diseases which affect children. The mortality from *apoplexy* was—males 4·0, females 3·5 in 10,000; from *paralysis*—males 3·1, females 3·5, in 10,000; the proportion of deaths from these maladies having been reversed in the sexes. To *chorea* (St. Vitus's dance), the deaths of 4 males and 20 females were ascribed; to *delirium tremens*, the deaths of 167 males and 15 females; *tetanus* (lock-jaw), 100 males and 29 females. The proportion in tetanus is 34 males to 10 females; but it is rarely an idiopathic disease, and men are exposed in almost a similar proportion to the injuries in which it originates. It will be observed that, to 10 females, 24 males died violent deaths.

Diseases of the *respiratory organs* produced 90,823 deaths, that is, a mortality of 6·0 in 1,000; while the annual mortality of the group in 1837 was 5·5 in 1,000, or 11 per cent. less. The mortality of *consumption* fell from 3·96 to 3·93 in 1,000; pneumonia, bronchitis, and pleurisy, rose from 6·93 to 1·38 (69 per cent.); asthma, from 2·5 to 3·8 (52 per cent.) 3·8 in 1,000 males, and 4·1 in 1,000 females, died of *consumption;* 11,691 males, and 9,488 females, died of inflammatory affections of the throat, larynx, air-tubes, lungs, and pleura. Consumption is 8 per cent. more fatal to females than to males. In point of fact, 27½ per cent. of the total deaths were due to diseases of the respiratory organs, and 18 per cent. to consumption; namely, 16·0 per cent. of the deaths of males, and 19·2 of the deaths of females.

Mr. Farr apostrophises stays very vigorously.

> "The higher mortality of English women by consumption may be ascribed partly to the in-door life which they lead, and partly to the compression, preventing the expansion of the chest, by costume. In both ways they are deprived of free draughts of vital air, and the altered blood deposits tuberculous matter with a fatal, unnatural facility. *Thirty-one thousand and ninety* English women died in one year of the incurable malady! Will not this impressive fact induce persons of rank and influence to set their countrywomen right in the article of dress, and lead them to abandon a practice which disfigures the body, strangles the chest, produces nervous or other disorders, and has an unquestionable tendency to implant an incurable hectic malady in the frame? Girls have no more need of artificial bones and bandages than boys."

2,032 males, and 1,530 females, are registered as having died of diseases of the heart and blood-vessels; but this is below the true number. Aneurism destroyed three times as many males (88) as females (31), the proportion observed in 1837.

Diseases of the *digestive organs* (unlike diseases of the chest) were less fatal than in the latter half of 1837; the mortality having declined from 1·4 to 1·3 in 1,000, or, including thrush, diarrhœa, dysentery and cholera, from 2·07 to 1·59 in 1,000. The deaths attributed to *teething* increased; in this, as in other respects, the heterogeneous cases under the head having a stricter affinity to diseases of the nervous system. *Stricture* of the intestinal tube is often caused by cancerous deposits, and hence affects more females than males. Fifty-one males and 117 females died of peritonitis, probably, in some instances, puerperal peritonitis; 318 males and 189 females of hernia. Exclusive of teething, 10,992 persons died of diseases of the stomach and bowels; 3 of diseases of the pancreas; 3,880 of diseases of the liver (including jaundice, 841); and 27 of diseases of the spleen. The 1,385 cases classed under "disease of the intestinal canal" comprised cases of chronic enteritis, gastritis, and dyspepsia, as well as some malignant diseases.

1,338 males, and 313 females, died of diseases of the urinary organs. The mortality of the former from stone and gravel was 4 in 100,000; of the latter, 0·5. The difference in the 7 heads is exaggerated by, but it cannot be exclusively attributed to, mechanical causes.

2,811 mothers died in childbirth and miscarriage, or about 5 in 1,000 cases, while the proportion in 1837 was 4 in 1,000.

The mortality from rheumatism, and diseases of the bones, joints, cartilages, tendons, and muscles, remained 1·4 in 10,000.

The deaths from affections of the integumentary system were comparatively few. 82 males and 18 females died of fistula, the proportion having been the same as in diseases of the urinary organs. Add to the class the acute diseases from the epidemics, with specific inflammations of the skin, and the mortality will be 2·01 in 1,000; while with typhus, and the epidemic diseases which affect the mucous membrane, the diseases of the intestinal canal were 2·58 in 1,000. The mortality from the two groups was 4·6 in 1,000; lower than in 1837, and 30 per cent. less than the mortality (6·0) from diseases of the respiratory organs.

Diseases of uncertain seat proved fatal to 21,871 males and 22,361 females. They include diseases in which the part was faultily left unspecified in the register, such as "inflammation;" or diseases which, like cancer, pervade several organs, and in which the distinction of parts is quite subordinate to the distinction in the essential nature of the morbid products. *Dropsy* was observed in 5,170 males, 7,172 females; *hæmorrhage* in 730 males, 488 females; *mortification* in 802 males, and 541 females; *malformations* in 93 males, 73 females.

Of *purpura* there died 27 females and 31 males. Under hydrocephalus, hydrothorax, ascites, ovarian dropsy, and dropsy, 22,428 deaths were registered; 10,734 males and 11,694 females.

*Sudden deaths* were cases in which inquests were held, but in which the causes of death were either not ascertained, or not intelligibly expressed. 3012 were registered; 1840 males and 1172 females. Sudden death is most frequently the result of internal hæmorrhage; but the subject is not and cannot be well understood, until *post mortem* examinations are more generally instituted in this country. Out of 10,000 males 6·5 died of sudden death or apoplexy, and 5·1 out of 10,000 females. With violent deaths, which are also generally sudden, the annual mortality of males was 18·1 in 10,000.—of females 9·6 in 10,000. Women have less chance of dying suddenly than men, in the proportion of 10 to 18.

12 per cent. of the deaths of females, and 10 per cent. of the deaths of males, were ascribed to *old age* and to natural decay.

The deaths of 125 males and 36 females were referred to *intemperance*. The difference in the proportion of the sexes is still greater in *delirium tremens*, sometimes designated drunkards' delirium; of which 167 males and 15 females died. 161 males and 46 females died of *gout*. The consumption of intoxicating liquors has increased faster than the population in the last 20 years; and the sale of spirits at a much more rapid rate than that of ale or wine, which can only be injurious when taken to excess. The average annual number of bushels of malt on which duty was paid in England was 25,834,345 in the 5 years 1820-4; 35,048,368 in 1834-8; in 1820-4 the quantity of wine returned annually for home consumption was 4,751,104 imperial gallons, in 1833-7 it was 6,461,886 gallons; in 1820-4, consumption duties were annually paid in England on 7,572,702 imperial gallons of proof spirits, which in 1834-8 had risen to 12,012,484 gallons; and the opium entered for home consumption rose in the same periods from

19,276 lbs. annually, to 33,482 lbs. The decennial rates of increase were for malt, 24; wine, 27; spirits, 39; opium, 53 per cent. For malt, the annual rate of increase was only 1·2 per cent. from 1810-4 to 1826-30; but the consumption rose rapidly under the Act to permit the sale of beer, and the annual rate of increase from 1827 to 1836 was 3·3 per cent. The consumption of wines rose from 4,681,357 gallons (1820-3) to 6,617,363 annually in 1824-8, when the duty was reduced from 9s. 1¼d. to 4s. 9¼d. a gallon. It then remained nearly stationary.

The 63 cases ascribed to starvation in 1837 have been since carefully investigated. 24 were infants that died for want of maternal nourishment; 12 died of inanition; 12 died from exposure to cold; 15 from the want of proper food, or from the want of the necessaries of life. The coroners were not the informants in the majority of instances. In 1838, the deaths of 126 males and 41 females, making 167 in all, were classed under starvation.

11,727 persons died violent deaths; of whom 8359 were males, 3368 females. They may be divided into voluntary and involuntary deaths. The number of registered suicides amounted to 1058 (males, 751, females, 307); and in many cases of individuals "found dead," the agent was not ascertained. The tendency to commit suicide increases up to the age of 60; the rate of increase is nearly 50 per cent. every 10 years.

It may be stated, as a general summary of the diseases, grouped according to their pathological characters, that 36,799 died from inflammations, 85,506 from specific inflammations, 19,122 from the terminations of inflammation, 15,125 from hæmorrhages, 2821 from carcinomatous diseases; 60,868 from tuberculous diseases; 2256 from disordered secretions, 2512 from depraved nutrition, 44,773 from disorders of the nervous system, 35,564 from old age, 11,727 from violent deaths.

Mr. Farr dwells with satisfaction on the fact that the results of the Registration in 1838 coincide very closely with those contained in the first report. We are delighted to transfer to our own pages the following high compliment to the country practitioners of Britain.

"The information as to the cause of death was obtained from the medical practitioners of the country, directly or indirectly; and the analysis of their observations, which is here presented, will show that they are not surpassed in intelligence or zeal for the promotion of science by the medical practitioners of any other country in Europe. May I venture to express a hope that they will contribute to render the registration of the causes of death still more accurate by giving, in every case which they attend, a written certificate, drawn up as nearly as may be upon the principles suggested in your first report."

### DISEASES OF TOWNS AND OF THE OPEN COUNTRY.

In the first half year of registration the difference between the diseases in a dense and scattered population was remarkable. But this might be accidental. Let us test it by the year 1838, which presented a great range in the temperature and the epidemic constitution.

The population in 1838 would be about 3,726,221 in the city districts, and about 3,539,908 in the counties. The city was probably to the rural population as 1·053 to 1·000; and to this extent (5 per cent.) the deaths in the counties should be augmented to render the mortality strictly comparable.

Besides the 70,410 persons who died equally in the dense and in the more scattered populations, there was an excess in the cities of 30,609 deaths; 9,970 from diseases of the epidemic class, 7,474 from diseases of the nervous system, 10,465 from diseases of the respiratory organs, and 3,144 from diseases of the digestive organs. The annual rate of mortality in the cities was 2·7, in the counties 2·0 per cent.; and the mortality in the cities 1·36 to 1·00 in the counties. The mean duration of life in the two sets of circumstances would differ nearly in the ratio of 37 years and 50 years.

In examining the special causes of death, three classes may be distinguished; one class which was exaggerated in cities to the highest pitch, a third class in which the mortality was nearly the same or in excess in the counties, and an intermediate class. To 1·00 deaths in the counties the deaths out of the *same amount of population* in the cities were by asthma, 3·80; erysipelas, 2·71; convulsions and teething, 2·57; cephalitis and hydrocephalus, 2·41; hydrophobia, 2·37; pneumonia, bronchitis, and pleurisy, 1·99; delirium tremens, 1·98; typhus, 1·88; small-pox, 1·73; heart-disease, 1·73; childbirth, 1·63: syphilis, 1·59; rheumatism, 1·58; gout, 1·55; hernia, 1·48; purpura, 1·46; sudden deaths, 1·45; liver disease, 1·45; hepatitis, 1·35; tetanus, 1·32. The excess of mortality in cities was less in the following cases: by consumption, 1·24; croup, 1·23; violent deaths, 1·17; stone, 1·11; mortification, 1·10; malformations, 1·07; apoplexy, 1·07; hæmorrhage, 1·02.—The mortality by the third class of causes was greater in the counties than in the cities: for the mortality to 1·00 in the counties was, in the cities, by paralysis, ·99; dropsy, ·99; jaundice, ·99; diabetes, ·97; cancer, ·92; hydrothorax, ·88; hematemesis, ·79; debility (frequently premature birth), ·75; atrophy, ·75; scrofula, ·46.

The same injuries and diseases are more fatal in cities than in the country. Parturition, from the frequency of puerperal fever, in town is 63 per cent. more fatal.

"If the mortality in the counties has been taken for unity, and all above it has been termed excess, it must not be understood to imply that less than 70,410 deaths may not be expected to occur out of a population of 3,539,908. The population of the counties, which have been held to represent the country, included the inhabitants of several cities. The mines of Cornwall caused many deaths; and any one who has visited the ill-ventilated dwellings of the poor, and is acquainted with their limited command of clothing, firing, and substantial food in agricultural districts cannot come to that conclusion. The minimum degree of sickness which a well-educated, affluent people would experience, and the years which they would number in the circumstances most favourable to health, are unknown; for the majority of the rich and middle classes whose lives have been observed, live principally in ill-constructed cities, and are exposed to the epidemics generated among their unhappier neighbours. It will be prudent therefore not to speculate upon a state of things of which the registers afford no examples, as it may sound paradoxical to fix more than fifty-five years for the average duration of human life; and it would not be practicable to suggest any means for improving by immediate measures the health of agricultural districts more effectual than the improvement of the cities in their centres, from which so many diseases radiate."

These reflections are extremely just, and extremely valuable. And they ought to attract the serious attention of our legislators. If the public health formed as anxious a subject of inquiry and solicitude, as political and party interests do, the public happiness would be materially increased.

Is, asks Mr. Farr, the excessive mortality of cities inevitable? He observes that it is only of late that such mortality has been fully proved and generally credited. It was scarcely known before the publication of the first report of the Registrar General, that the mean duration of life was from 25 to 30 years in the east districts, and from 40 to 50 years in the north and west districts of the metropolis. A fact so startling is calculated, when rightly considered, to inspire hope rather than dismay. We again quote Mr. Farr:—

"The first writers who established satisfactorily the high mortality of cities took a gloomy and perhaps fanatical view of the question. Cities were declared vortices of vice, misery, disease, and death; they were proclaimed 'the graves of mankind.' The population of the country, it was said, was drawn to them to be sacrificed; and those who entered left all hope behind, for no prospect of health in cities was beheld. Happily the further application of the methods which those eminent writers employed, and the facts which the registers furnish, enable us to analyse the causes of death in cities; and to show that while the mortality is increased as much as they stated, the apprehensions into which they were betrayed were ill-founded when applied to the future. There is reason to believe that the aggregation of mankind in towns is not inevitably disastrous. Health and life may be preserved in a dense population, provided the density be not carried beyond certain limits. Of this the nature of the causes to which the mortality is due, as well as the rapid improvement in the health of London within the last two centuries, is presumptive proof; and the favourable condition of several districts of the metropolis leaves little room for doubt on the subject."

Mr. Farr goes on to remark, that the primary objects to be kept in view are, the careful exclusion of all unnecessary animal and vegetable matter; the immediate removal of residual products; and the dilution of inevitable exhalations. The dead should no longer be buried where they are surrounded by crowded dwellings. Unwholesome manufactories should be excluded from densely-peopled districts. And there is assuredly no reason why thousands of cattle, sheep, horses, and animals of every kind—sometimes affected with epizootic diseases—should be gathered together in market-places within the city, or slaughtered in houses where the blood and offal can never be effectually removed. Public slaughterhouses have been erected in other places, without putting the butchers to any real inconvenience, or raising the price of meat. The supply of pure water, and a system of drains and sewers are other points which have attracted attention. The sewerage of the metropolis has been much improved; but the able Reports of the Parliamentary Committees show not only that there are still deplorable deficiencies and imperfections in the sewers, but that the unsatisfactory state of things is due, in no small degree, to imperfect laws, or to the machinery by which the laws are worked. If a survey were made of the districts of the metropolis, and the levels, the sewers, the drains, and the nuisances known to be pernicious were accurately laid down upon a map, it would be found to agree very remarkably with the table of relative mortality; and the construction of such a map would complete the view of the evil in all its details, and form the basis of a well-planned remedy. It will probably be found that the immense quantities of agricultural produce brought to London, and disgorged in the sewers, and the Thames, may be collected with less danger to the public health in distant reservoirs, filtered and returned, in the shape of manure, to the fields in the surrounding counties. The population is limited by the amount of subsistence, and the produce of the soil is limited by the quantity of this very organic matter which is so recklessly thrown into the wasting sea.

Wide streets, squares, and parks, with spacious houses, would render ventilation easy, and secure the dilution of poisonous emanations; but the ground is valuable, and building is dear in cities; hence there has been a constant and an unopposed tendency in landlords to accumulate the greatest number of houses on the least possible space in poor districts, and the families of artisans are driven to crowd in small, low, close rooms. The evils from this source are one of the contingencies of poverty and ignorance; they may, however, be met by opening, in the densest neighbourhoods, a certain number of wide streets, through which the collateral streets would be ventilated by fresh atmospheric currents. As information spreads among tenants, landlords will naturally render the districts in which their property lies healthy. Men will pay higher rents rather than expose themselves and their families to the risk of sickness and death. The landlords of the metropolis, at whose expense the improvements must be made, are deeply interested in its sanatory state; for every amelioration conducive to the health of the inhabitants, raises the value of houses, while the deterioration of the atmosphere must inevitably drive the wealthy out of town, and lead to the erection of residences in the country, which the facilities of travelling will every day render more accessible.

We of the metropolis, are deeply interested in whatever affects its sanatory state. But our country readers are scarcely less so. For, mutatis mutandis, what is true of the metropolis is true of the great provincial towns, and cities with their civilization and diseases are springing up throughout the length and breadth of England.

### Influence of Climate.

Mr. Farr remarks on the advantage, were it attainable, of determining this. He points out the steps he has taken in his calculations, but we need not specify them. Nor indeed can we insert the tables that embrace them. There is nothing on this head sufficiently satisfactory to detain us.

### Influence of Seasons.

Mr. Farr remarks, that reduction of temperature is one source of mortality —malarious exhalations are another. Winter, then, may be the most fatal to the inhabitants of a city on a favourable site, furnished with sewers, or to an agricultural population occupying a dry soil; and summer to the inhabitants of marshy districts, or of cities in which the refuse of organic matter is exposed to putrefaction.

The deaths registered in the metropolis in the winter quarter of 1838

amounted to 15,611 ; in spring, to 13,109 ; in summer, to 11,397 ; and in autumn to 1 ,581. Summer was the healthiest, winter the most fatal season : and this rule has prevailed in England since the beginning of last century. It is the reverse of the doctrines of the early English writers, and of the Greek and Roman physicians, summed up by Celsus in the aphorism : *Saluberrimum ver est: proxime deinde ab hoc, hiems : periculosior aestas: autumnus longé periculosissimus.* This order of salubrity, still observed in Rome, and in the towns on the shores of the Mediterranean, might be ascribed to the climate, if it had not formerly obtained in London, and other cities on nearly the same isothermal line. Julius Cæsar placed the *middle* of spring, summer, autumn, and winter, at the equinoxes and solstices; and according to this arrangement of the months, in which winter dates from November 11th, the relative mortality of the seasons in London was, winter 26, spring 16, summer 44, autumn 163, in five plague years of the seventeenth century ; and in five intercurrent years the mortality was, winter 17, spring 14, summer 15, autumn 24,—precisely the order laid down by Celsus.

The remarkable change in the relative mortality of the seasons in London will be best seen in the subjoined comparison.

The mortality of the seasons in the seventeenth century was deduced from the weekly bills of mortality; and the absolute mortality from the annual deaths, and from an enumeration of the population of the city of London, in 1631. It will be perceived at once, by this comparison, that the high rate of mortality in summer did not, in the seventeenth century, imply a low absolute rate of mortality in any season. The severities of winter were unmitigated, though they were thrown into obscurity by the plague. If the annual mortality of the metropolis had been the same in 1838 as it was in 1606-10, the deaths in the four seasons would have been—winter 26,200, spring 28,210, summer 39,670, autumn 37,960 : if it had been as high in July, August, September, as in the plague years, 307,950 might have been registered in three months, instead of 11,397. So much has the health of London improved : so much is life under human control!

And if such extension of existence in the metropolis *has* occurred, why may not equally striking advances still occur ? When we look around us, and observe the filth, the crowding, the want of drainage, of proper sanatory regulations, the destitution, and the intemperance that may still be found in the metropolis, and when we reflect both on the obviousness and practicability of the remedies, we may indulge the hope that the increase of this great city will bring with it a corresponding increase of the health, and consequently of the happiness of its inhabitants.

There was a peculiarity in the diseases, and a corresponding irregularity in the temperature, in the year 1838. At Chiswick in the vicinity of London, the mean temperature of winter (January 1st to March 31st) was 40°, spring 55°, summer 61°, autumn 45°, during the 10 years 1826-35; in 1838 the mean temperature of winter was 35°, spring 52°, summer 60°, autumn 44°. The mean temperature of the year was 47°·6, of the 10 years preceding, 50°·5. The mean temperature of January was 4° below the freezing point; while on an average of the 10 years, 1826-35, it was 4° above the freezing point. In the shade the thermometer fell 4½° below zero, or 14° lower than in 1826-35, when the minimum observed was 10°. The cold in London was less intense. The temperature of the four seasons at the apartments of the Royal Society, Somerset House, was 36°, 53°, 61°, 45°, of the year 48°·9. The thermometer did not fall lower than 11°.

The dryness of the seasons, measured at Chiswick with Mr. Daniel's dew point hygrometer, was 2°·2, 5°·7, 4°·7, 1°·3, in 1826-35, and 1°·9, 6°·1, 4°·2, 1°·0, in 1838. The moisture was above the average; yet the quantity of rain that fell was only 21½ inches, while the average quantity in 1826-35 was 24 inches.

The electric state of the atmosphere was not observed; but it is indicated by the deaths from lightning, which in the kingdom amounted to 24; in winter 1, spring 10, summer 11, autumn 2.

Professor Lindley has stated that "the winter of 1837-8 was in England more injurious to vegetation than any which has occurred in modern times, and it must be many years before its disastrous effects can be repaired, under the most favourable circumstances."

It is evident from these details that the registration of 1838 is well calculated to exhibit the influence of cold.

The cold increased the mortality in the metropolis from the following diseases to the greatest extent—paralysis, apoplexy, asthma, hydrothorax, bronchitis, pleurisy, pneumonia, influenza, diseases of the heart, &c. diabetes, dropsy, mortification, sudden deaths, and old age.

Cephalitis, hydrocephalus, and convulsiens were scarcely more fatal in winter than summer.

Consumption destroyed the greatest number in spring; but the excess of deaths may have been the result of the previous winter's cold. Males suffered from the disease in winter more than females.

Diseases of the digestive organs and some nervous affections prevailed in summer.

### Progress of Epidemics—Epidemic of Small-pox.

Mr. Farr observes that the registration has already yielded facts which are likely to throw light upon the propagation of epidemics. The deaths from small-pox in 324 divisions of the kingdom are exhibited separately in each of the ten quarters, from July 1st, 1837, to December 31st, 1839; a period comprising two winters, two springs, three summers, and three autumns.

The grand epidemic was composed of a succession of smaller epidemics; and, whether the commencement or the acme be considered, it is evident that the disease was not regulated by any cause, such as temperature; for, at the time that it was beginning in one district, it was at its height or was declining in another, placed in apparently the same general circumstances.

The following statement is as valuable as circumstantial.

When the Registration Act came into operation the epidemic of small-pox had commenced, and was rapidly advancing. It was raging at its height on the western side of the island. In Liverpool and West Derby 458 individuals perished, and were registered under small-pox, in the first three months. Bath, with a much smaller population, lost 154 lives; Leicester, 43; Shrewsbury 35. The epidemic prevailed in the south-west counties in autumn, extending to the districts around Bath, and then passing from Somersetshire to Devonshire, where it destroyed 131 lives in Exeter, and half as many more in the surrounding districts: and to Wiltshire, where 40 died in Calne, Marlborough, and Pewsey, 48 in Devizes, and 22 in Salisbury. It penetrated further into the country; 64 died in Wycombe, 72 in Wolverhampton, 57 in Blackburn, 99 in Wigan. The deaths from small-pox in Wales were tripled; 69 died in Wrexham, 85 in Abergavenny and Pontypool, 54 in Merthyr Tydfil. The hills and the valleys of Wales were traversed, and 711 victims were cut off, in the third quarter, the winter of 1838. The disease hovered over the Metropolis at the first: 22 died in Holborn, 10 in Whitechapel, 16 in St. George's, Southwark, 29 in Lambeth, 47 in Greenwich: the deaths from it were doubled in the second quarter; 753 perished in the winter of 1838. The surrounding districts were infected, Richmond, Kingston, Brentford, Staines, and Uxbridge; Dorsetshire, in the south-west, that had remained almost exempt, was visited; Weymouth, Bridport, Beaminster; then Sherborne, Dorchester, and Cerne; and Taunton that had been just left by dysentery, with Williton, Wellington, and Bridgewater, in Somersetshire. During the winter quarter (1838) not less than 121 died in Bristol and Clifton; 63 in Worcester, 36 in Dudley, 61 in Wolverhampton, 108 in Birmingham and Aston, 40 in Altrincham and Runcorn, 156 in Manchester and Salford. The small-pox mortality attained its maximum in the spring of 1838; the Metropolis saw 1,145 carried to premature graves; Surrey lost 83 by the malady; Kent, 132; Berkshire, 64; Wiltshire, 93; Somersetshire, 222; Gloucestershire, 142; Worcestershire, 89; Warwickshire, 107; Lancashire, 442; Yorkshire, 282; Durham, 88; Cumberland, 44; Monmouthshire, 82; Wales, 515. In three months, 4,489 deaths from small-pox were registered. The epidemic paused, either because its strength was exhausted or its victims failed; yet 3,685 fell under its hands in summer, 3,851 in autumn. On the Surrey side of the Thames, and at the west end of the Metropolis, the mortality attained the highest pitch in the summer and autumn of 1838; in the three last months of the year, 104 died of small-pox in the Westminster district. Ramsgate and Margate suffered severely. In the summer 76 died at Reading; in autumn, 62 in Ely, North Witchford, and Wisbeach, Cambridgeshire; 50 in Romford, Orsett, Billericay; 48 in Rochford and Maldon; 78 in Colchester, Essex; 95 in Ipswich; 48 in Plymouth; 180 in Manchester; 106 in Oldham; 197 in Leeds; 22 in Whitehaven; 38 in Westmorland.

In 1839 the epidemic reached Norfolk: in the spring, 127 were destroyed in Walsingham, Docking, Freebridge Lynn, and King's Lynn; 180 in Norwich, where 204 more died in the ensuing summer; the disease still raging in Essex and Suffolk, but with diminished violence. The North and the East Ridings of Yorkshire, Sunderland, Newcastle-upon-Tyne, and Tynemouth, were visited. The epidemic subsided on the eastern shore; and in the summer of 1839 only 1,533 died of small-pox in the kingdom: 65 cases of small-pox were registered in London. In the autumn of 1839 signs of a second epidemic appeared at Liverpool, Bath and other towns; the deaths in the kingdom rose to 1,730.

The epidemic destroyed more than 30,819 persons.

The annual rate of mortality from small-pox was 0·8 in 1,000. In the Metropolis 1·1; in Monmouthshire and Wales, 1·2 in 1,000.

Mr. Farr asks if the simple principle of contagion will explain the rapid propagation of the epidemic?

"Not exclusively: for the disease is always contagious, and a certain number of deaths are caused by it in all seasons, and in every county of England. The facilities of intercourse, and the frequency of contact with the sick, are not greater when the disease is increasing, or is at its height, than when it is stationary or declining. The fact that 2,513 died in the first period, 3,289 in the second period, and 4,242 in the third period, must therefore be accounted for, either by assuming that the disease had its origin in some spreading physical cause; that the contagious principle grew more virulent, and was conducted with greater facility by the atmosphere; that the susceptibility of the population increased; or, finally, that the tendency of the organization to fall into this peculiar pathological state augmented spontaneously. *Five* die weekly of small-pox in the Metropolis when the disease is not epidemic; and it will be recollected that the question is not to account for this rate of mortality, or for the five weekly deaths which may occur as other deaths occur, or be kept up by the uniform transmission of the disease from family to family. The problem for solution is,—Why do the five deaths become 10, 15, 20, 31, 58, 88, weekly, and then progressively fall through the same measured steps?"

He adds:—

"It may be contended that Manchester derived the small-pox from Liverpool. The intercourse between Liverpool and Manchester is perhaps more intimate than between any two towns in Europe. The epidemic broke out early in 1837, at Liverpool, and it appeared in Manchester later in the year; was it not then communicated by the population of Liverpool to the population of Manchester? It may have been so communicated. Epidemics are unquestionably transmitted from one place and people to another; but who will pretend to assert that, if all intercourse had been cut off between Manchester and Liverpool, quarantine had been established, and a *cordon sanitaire* had been drawn, such as was enforced in Prussia when cholera prevailed, that Manchester, with all the materials of disease in its streets, would never have suffered from an epidemic of small-pox. Isolated cases of small-pox existed all the while in Manchester; the seeds of an epidemic were there, and would not the causes which generated the epidemic in Liverpool have led to the same result in Manchester? At any rate, the evolution of the epidemic in Liverpool could not be traced to external contagion; and the problem remains for solution,—why did the deaths from small-pox rise so rapidly, that at last 418 individuals perished in three months, while the ordinary mortality in Liverpool and West Derby, from small-pox, is 27 in three months?"

The upshot of all such inquiries is—that we know nothing of the matter. It has been supposed, indeed, that epidemics are generations of infusoria. But they have not been seen, and why should they spring up as they do, and appear only at distant intervals? In the present state of knowledge, it is more useful to study the phenomena of epidemics, and determine their more certain and obvious laws. Such there appear, in this instance, to have been.

Mr. Farr terms the ten quarters in which the deaths were registered the ten periods, the first quarter the first period, the second the second period, &c. &c. The mortality increased up to the fourth registered period; the deaths in the first were 2,513, in the second 3,289, in the third 4,242; and it will be perceived at a glance that these numbers increased very nearly at the rate of 30 per cent. For, multiply 2,513 by 1·30, and it will become 3,267; multiply 3,267 by 1·30, and it will become 4,248. The rate of increase is retarded at the end of the third period, and only rises 6 per cent. in the next, where it remains stationary, like a projectile at the summit of the curve which it is destined to describe.

The decline of the epidemic was less rapid than its rise, and the mortality was somewhat greater in the autumns of 1838 and 1839 than in the summers. But, by taking the mean of the deaths in the third and fourth period, the mean of the deaths in the fourth and fifth period, &c., &c., a regular series of numbers is produced.

Deaths observed in the decline of the Epidemic.

| 1 | 2 | 3 | 4 | 5 | 6 | 7 |
|---|---|---|---|---|---|---|
| 4,365 | 4,087 | 3,767 | 3,416 | 2,743 | 2,019 | 163 |

Deaths in a regular series.

| 1 | 2 | 3 | 4 | 5 | 6 | 7 |
|---|---|---|---|---|---|---|
| 4,364 | 4,147 | 3,767 | 3,272 | 2,716 | 2,156 | 1,635 |

The mortality from small-pox was greater in the metropolis than in all the other parts of England; and the rate of increase in the second, third, and fourth periods was 1·50, the deaths having been 506, 753, and 1,145. The rate of increase in the first and second periods was 1·97, the deaths were 227 and 506.

The decline of the epidemic in the metropolis is shown by the following numbers:—

Metropolis.

| | 1 | 2 | 3 | 4 | 5 |
|---|---|---|---|---|---|
| 1. Mean quarterly deaths registered | 1,103 | 959 | 611 | 240 | 91 |
| 2. Calculated series .. .. .. .. | 1,103 | 967 | 611 | 278 | 91 |

The rates vary with the density of the population, the numbers susceptible of attack, the mortality, and accidental circumstances; so that, to obtain the mean rates applicable to the whole population, or to any other portion of the population, several epidemics should be investigated. It appears probable, however, that the small-pox increases at an accelerated and then a retarded rate; that it declines first at a slightly accelerated, then at a rapidly accelerated, and lastly at a retarded rate, until the disease attains the minimum intensity, and remains stationary.

